# Supplementary material for: Preparation of Polyimide/Ionic Liquid Hybrid Membrane for CO2/CH4 Separation
Source: Polymers (Basel). 2024 Jan 31;16(3):393. doi: 10.3390/polym16030393 (PMC10856979; doi:10.3390/polym16030393)
Supplement: Supplementary file 1 [file polymers-16-00393-s001.zip › polymers-2651261-supplementary.pdf]

Supporting Information

Preparation of Polyimide/Ionic Liquid Hybrid Membrane for CO_2/CH_4 Separation

Yanqing Qu, Shijun Zhao, Hongge Jia*, Shuangping Xu, Mingyu Zhang, Guoliang Geng, Xiaoyu Du.

College of Materials Science and Engineering, Qiqihar University, Qiqihar, Heilongjiang 161006, China

*Correspondence to: jiahongge@qqhru.edu.cn

Table of Contents

Section A. Supporting Scheme S2

Section B. Supporting Tables S2

Section C. Supporting References S4

Section A. Supporting Scheme

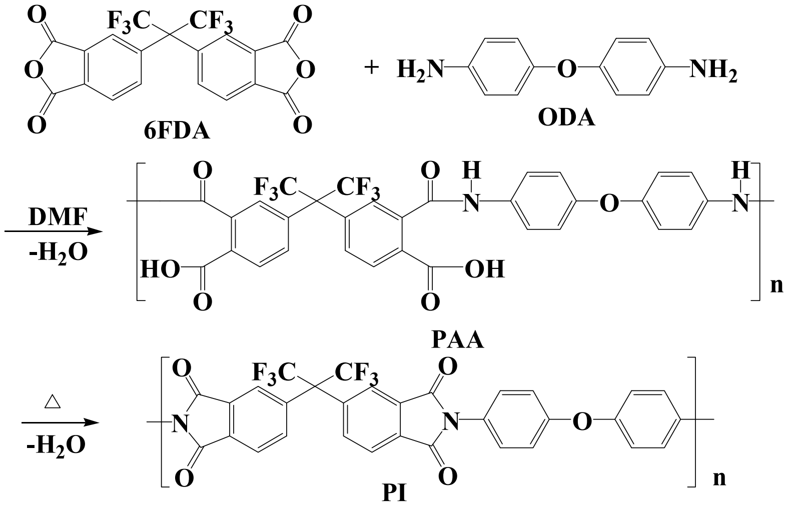

Scheme S1. Synthesis route of PI membrane.

ODA (0.40 g, 2.00 mmol), 6FDA (0.91 g, 2.04 mmol), and DMF (5.50 mL) were added to a three-necked flask and stirred for 4-5 hours to ensure sufficient reaction. The ratio of ODA to 6FDA was 1:1.02 and there was a small excess of anhydride (6FDA) because of the ease with which it can be hydrolyzed when exposed to air. To ensure the obtained polyimide reacted following the content we set, the excess of 6FDA was 2 mol%. After stirring, the polymer solution was placed and defoamed to obtain a polyamic acid solution (PAA).

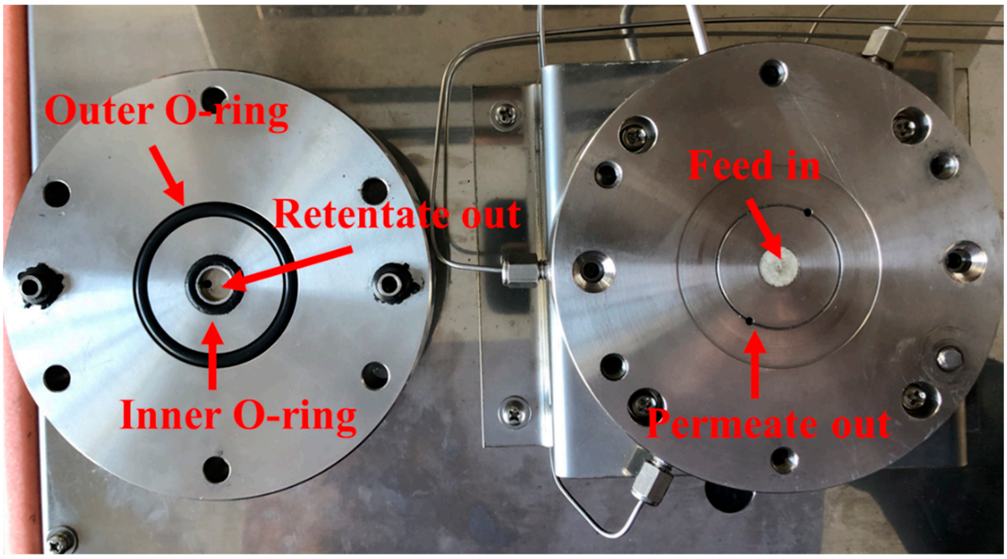

Figure S1. Schematics of experimental setup of the gas separation measurement.

Section B. Supporting Tables

Table S1. Mechanical properties of PI and PI/IL_n (x %) membranes.

NO.	Membranes	σs [a](MPa)	εb [b] (%)	TS [c](MPa)	E [d] (×10^3) (MPa)
1	PI	27±1	5±1	44±4	0.80
2	PI/IL_1 (5 %)	88±4	14±2	125±4	0.82
3	PI/IL_1 (10 %)	105±1	16±0	127±4	0.91
4	PI/IL_1 (15 %)	109±5	21±1	131±2	1.65
5	PI/IL_1 (20 %)	95±1	6±2	110±1	0.60
6	PI/IL_2 (5 %)	73±2	5±0	86±1	0.71
7	PI/IL_2 (10 %)	101±4	9±1	118±1	1.24
8	PI/IL_2 (15 %)	112±4	20±3	166±3	1.96
9	PI/IL_2 (20 %)	83±1	6±1	93±1	1.51
10	PI/IL_3 (5 %)	88±2	9±5	98±0	0.78
11	PI/IL_3 (10 %)	99±1	16±1	133±2	1.52
12	PI/IL_3 (15 %)	139±4	21±7	151±3	2.09
13	PI/IL_3 (20 %)	119±1	11±4	95±2	1.24
14	PI/IL_4 (5 %)	61±2	6±0	95±2	1.43
15	PI/IL_4 (10 %)	83±5	9±0	130±4	1.72
16	PI/IL_4 (15 %)	120±11	10±1	144±6	1.63
17	PI/IL_4 (20 %)	88±5	6±1	95±0	1.62

[a] Yield strength (σs), [b] Elongation at break(εb), [c] Tensile strength (TS), [d] Modulus of elasticity (E).

Table S2. Permeability and selectivity PI and PI/IL_n (x %) membranes.

NO.	Membranes	P_{CO_2}(Bar)[a]	P_{CH_4}(Bar)[a]	α(P_{CO_2}/P_{CH_4})
1	PI	09.56	0.13	073.54
2	PI/IL_1 (5 %)	20.33	0.26	078.19
3	PI/IL_1 (10 %)	19.88	0.21	094.67
4	PI/IL_1 (15 %)	11.44	0.10	114.40
5	PI/IL_1 (20 %)	12.03	0.15	080.20
6	PI/IL_2 (5 %)	21.03	0.27	077.89
7	PI/IL_2 (10 %)	20.04	0.25	080.16
8	PI/IL_2 (15 %)	12.19	0.08	152.38
9	PI/IL_2 (20 %)	13.79	0.16	086.19
10	PI/IL_3 (5 %)	22.79	0.28	081.39

11	PI/IL3 (10 %)	21.33	0.25	085.32
12	PI/IL3 (15 %)	16.25	0.09	180.55
13	PI/IL3 (20 %)	19.27	0.22	087.59
14	PI/IL4 (5 %)	23.06	0.36	064.06
15	PI/IL4 (10 %)	22.84	0.33	069.21
16	PI/IL4 (15 %)	11.79	0.16	73.69
17	PI/IL4 (20 %)	19.71	0.32	061.59

[a] 1 Barrer = 10^{-10} cm^3 (STP)*cm $cm^{-2}s^{-1}$ $cmHg^{-1}$

Table S3. Dissolution coefficient and diffusion coefficient of PI and PI/ILn (x%) membranes.

NO.	Membranes	SCO_2[a]	SCH_4[a]($\times10^{-3}$)	DCO_2[b]($\times10^{-10}$)	DCH_4[b]($\times10^{-10}$)
1	PI [1]	0.14	2.85	67.75	45.58
2	PI/IL1 (5 %)	0.36	21.30	56.47	12.21
3	PI/IL1 (10 %)	0.51	16.70	38.98	12.57
4	PI/IL1 (15 %)	0.62	8.82	18.45	11.34
5	PI/IL1 (20 %)	0.63	14.10	19.10	10.64
6	PI/IL2 (5 %)	0.43	20.70	48.91	13.04
7	PI/IL2 (10 %)	0.76	14.60	26.39	17.12
8	PI/IL2 (15 %)	0.80	7.86	15.23	10.18
9	PI/IL2 (20 %)	0.90	37.10	15.32	4.31
10	PI/IL3 (5 %)	0.50	18.30	45.58	15.30
11	PI/IL3 (10 %)	0.82	11.00	26.01	22.73
12	PI/IL3 (15 %)	0.88	7.63	18.42	11.79
13	PI/IL3 (20 %)	0.77	28.76	25.03	7.65
14	PI/IL4 (5 %)	0.52	35.70	44.35	10.08
15	PI/IL4 (10 %)	0.79	20.97	28.91	15.74
16	PI/IL4 (15 %)	0.65	30.53	18.14	5.24
17	PI/IL4 (20 %)	0.20	15.22	98.55	21.02

[a] cm^3(STP)*$cm^{-3}cmHg^{-1}$, [b] cm^2 s^{-1}

Section C. Supporting References

(1) Tanaka, K.; Islam, N.; Kido, M.; et al. Gas permeation and separation properties of sulfonated polyimide membranes. *Polymer*. **2006**, 47, 4370-4377.
